# Use of a Medical Communication Framework to Assess the Quality of Generative Artificial Intelligence Replies to Primary Care Patient Portal Messages: Content Analysis

**DOI:** 10.2196/71966

**Published:** 2025-07-31

**Authors:** Natalie S Lee, Nathan Richards, Jodi Grandominico, Robert M Cronin, Amanda K Hendricks, Ravi S Tripathi, Daniel E Jonas

**Affiliations:** 1 Department of Internal Medicine, College of Medicine, The Ohio State University, 2050 Kenny Rd, suite 2400, Columbus, OH, 43221, United States, 1 614-814-1361; 2 Health System Informatics, The Ohio State University Wexner Medical Center, Columbus, OH, United States

**Keywords:** communication, artificial intelligence, primary care, electronic health record, patient portal, health communication

## Abstract

**Background:**

There is growing interest in applying generative artificial intelligence (GenAI) to respond to electronic patient portal messages, particularly in primary care where message volumes are highest. However, evaluations of GenAI as an inbox communication tool are limited. Qualitative analysis of when and how often GenAI responses achieve communication goals can inform estimates of impact and guide continuous improvement.

**Objective:**

This study aims to evaluate GenAI responses to primary care messages using a medical communication framework.

**Methods:**

This was a descriptive quality improvement study of 201 GenAI replies to a purposively sampled, diverse pool of real primary care patient messages in a large midwestern academic medical center. Two physician reviewers (NSL and NR) used a hybrid deductive-inductive approach to qualitatively identify and define themes, guided by constructs from the “best practice” medical communication framework. After achieving thematic saturation, the reviewers assessed the presence or absence of identified communication themes, both independently and collaboratively. Discrepant observations were reconciled via discussion. Frequencies of identified themes were tallied.

**Results:**

Themes in strengths and limitations emerged across 5 communication domains. In the domain of *rapport building*, expressing respect and restating key phrases were strengths, while inappropriate or inadequate rapport building statements were limitations. For *information gathering*, questions that built toward a plan or elicited patient needs were strengths, while questions that were out of place or redundant were limitations. For *information delivery*, accurate content delivered clearly and professionally was a strength, but delivery of inaccurate content was an observed limitation. GenAI responses could *facilitate next steps* by outlining choices or providing instruction, but sometimes those next steps were inappropriate or premature. Finally, in *responding to emotion*, strengths were that emotions were named and validated, while inadequate or absent acknowledgment of emotion was a limitation. Overall, 26.4% (53/201) of all messages displayed communication strengths without limitations, 27.4% (55/201) had limitations without strengths, and the remaining 46.3% (93/201) had both. Strengths outnumbered limitations in *rapport building* (87/201, 43.3% vs 35/201, 17.4%) and *facilitating next steps* (73/201, 36.3% vs 39/201, 19.4%). Limitations outnumbered strengths in the remaining domains of *information delivery* (89/201, 44.3% vs 43/201, 21.4%), *information gathering* (60/201, 29.9% vs 43/201, 21.4%), and *responding to emotion* (7/201, 8.5% vs 9/201, 4.5%).

**Conclusions:**

GenAI response quality on behalf of primary care physicians and advanced practice providers may vary by communication function. Expressions of respect or descriptions of common next steps may be appropriate, but gathering and delivering appropriate information, or responding to emotion, may be limited. While communication standards were often met, they were also often compromised. Understanding these strengths and limitations can inform decisions about whether, when, and how to apply GenAI as a tool for primary care inbox communication.

## Introduction

Primary care is in crisis. The demand for services far exceeds supply, while the daily burden of responsibilities is increasingly untenable for primary care physicians and advanced practice providers (PCPs) [1]. Patient messages submitted to their providers through electronic health record (EHR) portals are a well-recognized burden for clinicians [2-4], especially in primary care [5,6]. Nationally, initiatives to address and mitigate these sources of PCP burden and burnout have become a priority, including for the National Academy of Medicine [7], the American Medical Association [8], the US Surgeon General [9], and the Agency for Healthcare Research and Quality [10].

A novel potential strategy is the adoption of generative artificial intelligence (GenAI) tools to draft replies to patient messages on behalf of PCPs [11]. GenAI technologies have demonstrated capacity to pass medical board examinations [12] and make accurate diagnoses [13], and they are already transforming day-to-day clinical practice. Thoughtful application of GenAI tools has the potential to improve many deficiencies of health care delivery for both patients and PCPs. However, the “when” and “how” of applying GenAI to respond to primary care messages is still in its formative period, with a high price of investment and uncertain impact. Viable solutions must reduce PCP burden while maintaining or enhancing patient care quality. Thoughtful evaluations in this early period are critical, both to inform decisions about investing in these tools and to direct their development and application in ways that contribute genuine value to primary care.

A small but rapidly growing body of reports from early adopters examines GenAI responses to real patient messages submitted to their care teams. Reported evaluation metrics include draft “use,” defined as the human author’s decision to start the response with at least a portion of the GenAI draft, and physician work metrics, such as time required to read and write messages [14-16]. However, draft use is a poor indicator of meaningful impact on PCP burden or patient care, while physician work metrics have been largely unaffected [14,15]. Surveys and brief qualitative feedback have provided further insights, with data suggesting variable perceptions of GenAI draft quality and mixed impact on workload, ranging from helpful to unhelpful or even increasing work due to editing burden [15-17]. More data are needed to inform our understanding of the potential role for GenAI drafts in primary care, and in what ways the technology could be better leveraged to optimize its use.

Frameworks for medical communication are widely accepted and taught, with a consensus on “best practice” for high-quality communication [18]. Ideal automation would ensure that standards for communication were consistently met, and such a tool would invariably be helpful to PCPs while maintaining or enhancing patient care. However, no prior studies have applied a medical communication framework to systematically evaluate GenAI draft quality or the consistency of its quality. Another limitation of prior studies is that GenAI messages on behalf of multiple human author types, such as medical assistants, nurses, and physicians and advanced practice providers, were mixed, as were specialty contexts such as primary care versus other specialties. Greater specificity in evaluation is important because the nature of messages sent by patients and the expected information from the responses differs across these diverse author types and contexts.

This quality improvement study addresses these gaps by applying the “best practice” communication framework to systematically identify and quantify themes in communication quality among GenAI responses to diverse primary care patient messages. We focus on messages to, and drafts on behalf of, PCPs, allowing us to apply a uniform standard of evaluation and collect more precise insights for the *where* and *how* of GenAI response tools in primary care.

## Methods

### Study Design

We conducted a thematic analysis of 201 unique GenAI messages using a hybrid deductive-inductive approach, guided by the “best practice” communication framework. The 201 GenAI messages were generated in response to a diverse pool of primary care patient messages, using prompts that had undergone multiple prior rounds of optimization.

We first describe the prestudy context, including how the study sample of GenAI responses to patient messages was derived and how GenAI prompts were optimized prior to thematic analysis of its output. We then describe the methods used in the qualitative analysis of the 201 unique GenAI messages.

### Prestudy Context: GenAI Response Pilot in Primary Care

At our institution, a large midwestern academic medical center, we piloted the GenAI draft tool offered by our EHR vendor, Epic, from September 14, 2023, through April 30, 2024, which at that time was based on GPT 3.5. The tool is used to generate a response to a patient-submitted message; the message recipient can choose to use, edit, or ignore the draft reply in responding to the patient. The purpose of our pilot was to gain sufficient experience with GenAI responses to make an informed decision about institutional investment in the tool for use by PCPs. The pilot team included 6 PCP volunteers and a cohort of analysts from the medical center’s health system informatics team.

Aiming to uncover both the strengths and limits of the tool’s capabilities, the pilot team took several steps to maximize variation in the pool of patient messages used to test the model. The PCP volunteers were diverse across a range of factors that could influence patient message variation: 3 from family medicine and 3 from general internal medicine, 4 women and 2 men, an equal number of White and non-White participants, and a broad span of clinical experience, from 5 years to several decades in independent practice (median 18 years). Analysts pulled the most recent 400 patient messages submitted to the participating PCPs, distributed evenly across them. It was possible for unique patients to contribute multiple messages to the pool. From this pool, they manually curated a final sample of 200 messages selected for diversity in content, language, and complexity.

GenAI output is sensitive to the wording of prompts, or instructions, provided, and the creation of appropriate prompts for the task at hand is critical for optimizing the quality of the GenAI response [19,20]. Thus, the pilot team sought to optimize GenAI prompts through multiple rounds of prompt engineering, which is the term for optimizing instructions for the GenAI model. Our informatics analysts served as the primary prompt engineers. Standards for prompt engineering have not yet been developed in health care, but our prompt engineers used all resources available through Epic and publicly available external resources. Starting with the Epic-suggested prompt, prompt engineering was further guided by examples of prompts used at other institutions and attendance at multiple prompt engineering “office hours,” during which prompt engineers from health care organizations across the country shared experiences, ideas, and insights.

Prompt engineering rounds were iterative. For each new or revised prompt, the participating PCPs reviewed the GenAI draft responses, paired with the corresponding patient messages in adjacent columns on a spreadsheet and no other chart information. Anywhere from 30 to the full set (n=200) of drafted responses was reviewed for each round, with larger quantities in earlier rounds and smaller quantities in later rounds to improve review efficiency. Analysts used subjective assessment to ensure that each round was based on a diverse subset of patient messages. Each round concluded with a virtual meeting among participating PCPs and health system analysts to discuss their perceptions of GenAI draft messages, considering tone, information, and helpfulness of the reply. Combining this feedback with insights from vendor resources (office hour notes and prompt library), key areas for changes to the prompt were identified for the next round of prompt engineering.

**Figure 1.**
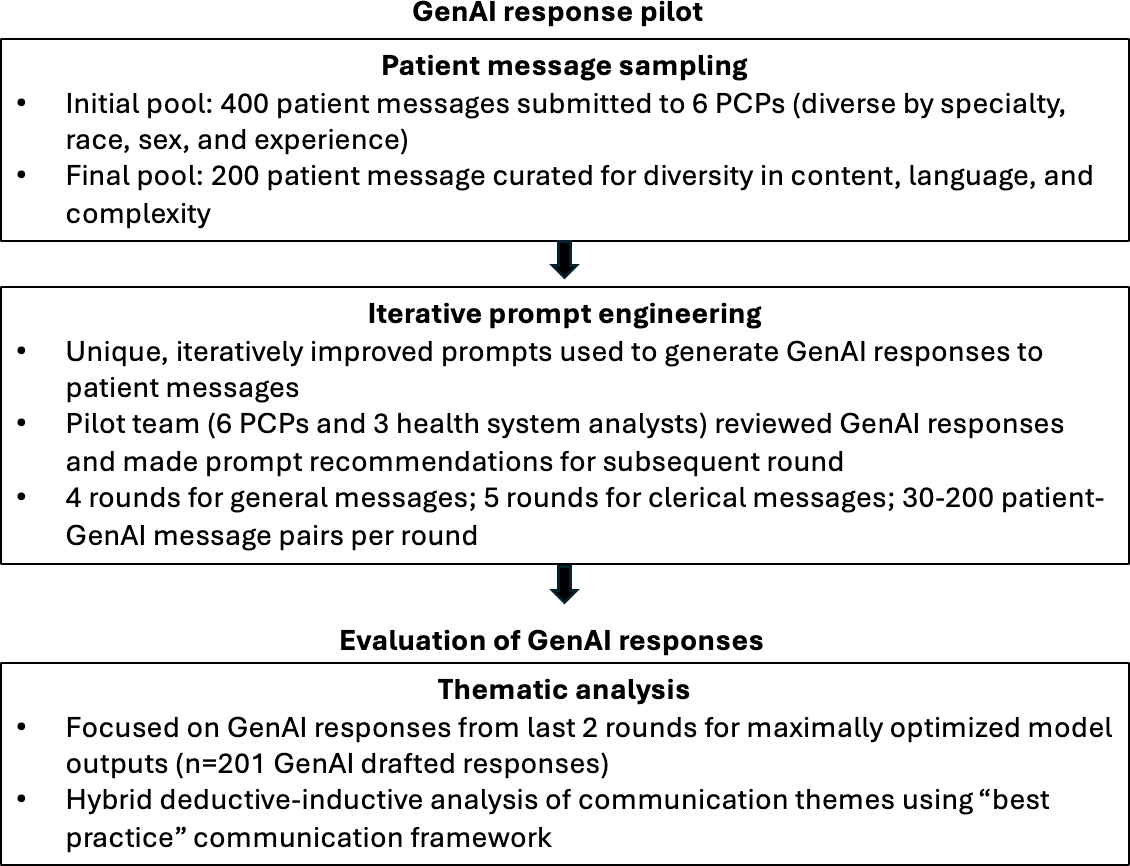
Flowchart depicting pilot activities and the subsequent thematic analysis of artificial intelligence–generated responses. GenAI: generative artificial intelligence; PCPs: primary care physicians and advanced practice providers.

For further optimization, prompt engineering rounds were conducted separately for different categories of messages, as identified by Epic’s proprietary message filter (Figure 1). *Clerical messages* pertained to requests for paperwork, letters, medications, and test results; *general messages* were all other message types. Through this process of interactive and iterative discussion, 5 rounds of prompt engineering for general patient messages and 4 rounds for clerical messages were completed between November 1, 2023, and May 1, 2024. Decisions not to pursue additional rounds of prompt engineering were based on multiple factors, including minimal perceived difference in AI responses between later rounds and time and resource restraints.

Across prompt engineering rounds, the GenAI model had limited information from patients’ medical records. Patients’ preferred names and pharmacies were always accessible. For general messages, patients’ age, allergies, and all future appointments were also accessible. For clerical messages, outpatient medication lists, employer names, and test results for the last 2922 hours (approximately 3 months) were also accessible. These were decisions based upon institutional patient privacy concerns, costs per token sent to the model, and model time interval limits.

During the pilot, 2 physicians withdrew participation early, citing a lack of enthusiasm after the first few rounds of prompt engineering. At the end of the pilot, the remaining physicians unanimously voted against adoption. Their feedback was that similar functionality could be achieved through existing EHR tools, while reviewing and editing GenAI responses increased cognitive burden.

### Evaluation of GenAI Responses

Upon completion of the pilot, we sought to more systematically assess and convey the quality of GenAI draft responses through the application of the “best practice” communication framework [18]. We followed reporting guidelines from the Standards for Quality Improvement Reporting Excellence [21], recommended by the Enhancing the Quality and Transparency Of health Research network [22].

GenAI draft messages from the last 2 rounds of prompt engineering in each message category (n=201 total) (n=139 for general, n=62 for clerical) were included in the analysis, as these represented messages that had been generated using maximally optimized prompts, through several prior rounds of prompt engineering. The number of GenAI drafts reviewed exceeded the number of unique patient messages in the pool because, as described in the “Prestudy Context” section, patient messages could be randomly reused for subsequent prompt engineering rounds. Two primary care physician reviewers (1 who had participated in the prompt engineering pilot and 1 who had not) reviewed all 201 messages. As in the initial pilot, GenAI drafts were presented to reviewers in a column adjacent to corresponding patient messages and other clinical information (eg, notes, laboratory data, medications, etc) was not included. Thus, reviewers did not have more contextual information than the GenAI model and assumed the information it was accessing (medication lists, future appointments, etc) was accurate.

### Thematic Analysis

A hybrid deductive-inductive analytic approach was used to identify themes and create a comprehensive codebook. We established five overarching domains of communication aligned with the domains of the “best practice” communication framework: (1) *rapport building*, (2) *information gathering*, (3) *information delivery*, (4) *facilitating next steps*, and (5) *responding to emotions*. A final sixth domain of the “best practice” framework, “enabling patient behavior,” was folded into the “facilitating next steps” domain. These 5 domains served as the foundational categories for organizing the data.

Incorporating an inductive approach, we then used observations from the GenAI response to identify and define specific themes within each domain. Through iterative coding and discussion between reviewers, we examined how emergent themes related to original constructs within the “best practice” communication framework. We thus refined and contextualized each domain with themes grounded in our observations of the GenAI responses, while remaining theoretically informed.

We identified and refined themes until at least half of the dataset had been reviewed and there was mutual agreement that thematic saturation had been achieved. We stratified this process by message type (clerical vs general) in case meaningful differences in themes emerged between them; thus, we reviewed and analyzed 70 GenAI drafts for general messages and 32 for clerical messages (103 messages combined) collaboratively to develop and refine the codebook [18]. Ultimately, because themes were similar between message types, a uniform codebook was applied to all messages. The final codebook identified and defined binary categories—strengths versus limitations—of themes within each domain, resulting in 10 unique message categories.

### Tabulation of Themes and Statistical Analyses

Next, we applied the codebook to assess how often the identified communication themes emerged across the entire sample of GenAI draft responses (n=201). All code categories were independent and not mutually exclusive, so that a message could be coded as demonstrating both strength and shortcoming in a particular communication domain (eg, a message could have both strengths and limitations in the “rapport building” domain). However, per message, each category (strength or limitation within the 5 domains) was counted only once per message (eg, a strength in “fostering relationship” could be marked only once for a given message, even if additional examples of strengths in this domain existed elsewhere in the GenAI draft). We did not require all communication domains to be present in each draft; rather, we assessed strengths and limitations only when the message touched on a relevant domain. After assessing the first half (n=70 for general, n=32 for clerical, and n=103 combined) collaboratively to confirm interrater coding alignment, the remaining messages (n=69 general and n=30 clerical) were reviewed and coded by each reviewer independently. Agreement between reviewers for the latter batch was assessed using Cohen κ [23,24], and all discrepancies were reconciled via discussion, guided by the tenets of the “best practice” communication framework. The final tally of communication themes was based solely on reconciled observations.

Within the domain of “information delivery,” under the category defined as “limitations,” we identified multiple granular subcategories, representing distinct types of limitations specific to this domain (4 subcategories for general messages and 5 subcategories for clerical messages). While these subcategories were ultimately collapsed into a single coherent definition of “limitations” in the final codebook, we retained them during analysis due to their potential to yield valuable and actionable insights. Both reviewers independently assessed these subcategories whenever a limitation in this domain was coded. Given the exploratory nature of these subcategories, we did not formally assess interrater agreement or reconcile discrepancies at the subcategory level. We tallied the frequency of each limitation subtype, counting observations if marked by at least 1 reviewer.

Finally, each reviewer rated whether they were likely to use at least a portion of the GenAI draft in their own response to the patient message. For drafts that were assessed to be usable, reviewers assessed whether major edits versus minor to no edits would be needed. If the reviewer assessed that no portion of the GenAI draft message would be retained, the GenAI draft was assessed as “would not use.” We did not reconcile these assessments, acknowledging the role of individual physician preference.

### Ethical Considerations

The Ohio State University institutional review board determined that this activity met criteria for quality improvement and was thus exempt from human subject ethics review. Informed consent was also waived. Study data were not deidentified, and protective measures included keeping all data on secure servers that met the highest standards for restricted institutional data, as well as limiting access to those directly involved with the study. No compensation was provided to individuals whose data were included.

## Results

We identified strengths and limitations of GenAI responses across 5 communication domains (Table 1). In the domain of *rapport building*, observed strengths were that responses expressed respect and restated key phrases, while observed limitations were that rapport building phrases seemed inappropriate, inadequate, or were absent. For *information gathering*, observed strengths were that GenAI-posed questions elicited patient needs and contributed to clinical decision-making, while limitations were observed when the questions posed were inappropriate or redundant. For *information delivery*, observed strengths were that responses provided accurate information professionally, while limitations were observed when the information was inaccurate or unsuited for the context. In *facilitating next steps*, GenAI responses not only successfully outlined choices or next steps but also demonstrated limitations by moving to next steps prematurely or suggesting inappropriate next steps. For *responding to emotion*, successful responses named and validated emotions conveyed in patient messages, while limitations in this domain were observed when there was inadequate or absent acknowledgment of emotion. These definitions were consistent for general and clerical messages.

The Cohen κ measure of interrater reliability of assessments was 0.55 for general messages and 0.59 for clerical, both suggesting moderate agreement [23,24]. Overall, 26.4% (53/201) of all messages exhibited only strengths in communication, while 27.4% (55/201) of messages exhibited only limitations in communication with no identifiable strengths (Table 2). The remaining 46.3% (92/201) of messages had a mix of both strengths and limitations in communication domains.

Strengths were more frequently observed than limitations in the domains of rapport building and facilitating next steps (Multimedia Appendix 1). Half of all replies to general messages and 25% of replies to clerical messages contained appropriate expressions of respect. Meanwhile, nearly 40% of replies to clerical messages and a third of replies to general messages outlined reasonable next steps. For the remaining domains of information gathering and delivery, as well as responding appropriately to patient emotion, limitations were more frequently observed than strengths.

In exploratory analysis, there were multiple identifiable subcategories of limitations in information delivery. The reasons were that the patient’s informational need was not addressed (34/56, 60.7%), the response contained inaccurate information about clinical workflow (21/56, 37.5%), the response was simply wrong (11/56, 19.6%), and, rarely, because the delivery was unprofessional (1 message). Similar subcategories were observed for clerical messages with inadequate information delivery: informational need not addressed (24/33, 72.7% of messages), inaccurate assumptions about workflow (11/33, 33.3%), and information was wrong (10/33, 30.3%). Two additional subcategories for clerical messages were that the answer made inappropriate assumptions about the clinical context (8/33, 24.2%), and that the information provided did not appropriately account for shared understanding as implied in the patient’s message (5/33, 15%).

Finally, both physicians also determined whether each would use the GenAI draft. On average, the physicians assessed that 56.5% of messages of general messages could be used (53.2% for reviewer 1 and 59.7% for reviewer 2), with “fair” interrater agreement about which messages were usable (Cohen κ=0.257). Of those messages being used, 25.7%-65.1% (mean 45%, SD 27.9%) would require major edits. On average, for clerical messages, physicians assessed that they would use 45.2% of clerical message draft replies (46.8% for reviewer 1 and 43.5% for reviewer 2). Interrater agreement on message usability was almost perfect (0.81). Of messages being used, 27.6%-37.0% (mean 32.3%, SD 6.6%) would require major editing.

**Table 1.** Results of thematic analysis: medical communication strengths and limitations observed in generative artificial intelligence drafts.

Domain	Strength	Examples	Limitation	Examples
Rapport building	Expresses respect; repeats key phrases back.	“I appreciate your update.” “It’s great to hear that…”	Inappropriate or inadequate rapport building.	Repeats phrases back to the patient that miss the essence of the question; generic “I understand you are seeking X” does not suit the context.
Information gathering	Questions build toward plan, elicit patient needs.	Asks clinically appropriate follow-up questions about new symptoms.	Questions are out of place or redundant.	Questions focus narrowly on a single problem that is not the main clinical issue; the patient’s message clearly suggests that further questioning is unnecessary.
Information delivery	Appropriate content delivered professionally, avoiding jargon.	Provides accurate information about what to expect with a medical test.	Inappropriate or unsuitable content.	Patient asks about kidney donation, but the response provides information relevant to blood donation.
Facilitating next steps	Outlines choices, leads to appropriate next steps.	Responds affirmatively to patient request for blood test and provides appropriate logistical instructions.	Inappropriate or premature next steps.	Reply inappropriately complies with patient request for a specific blood test when more history and evaluation are indicated.
Responding to emotion	Names and validates emotions.	Patient expresses anxiety, and reply acknowledges, “It’s natural to feel anxious about this.”	Acknowledgment of emotion is inadequate or absent.	The patient expresses a long-standing emotional experience related to a condition; the reply does not acknowledge the patient’s emotion.

**Table 2.** Frequency of domain strength and limitations by message type.

Domain	General (n=139), n (%)		Clerical (n=62), n (%)	
	Strength	Limitation	Strength	Limitation
Rapport building	71 (51.1)	27 (19.4)	16 (25.8)	8 (12.9)
Information gathering	42 (30.2)	52 (37.4)	1 (1.6)	8 (12.9)
Information delivery	38 (27.3)	56 (40.3)	5 (8.1)	33 (53.2)
Facilitating next steps	47 (33.8)	26 (18.7)	26 (41.9)	13 (21.0)
Responding to emotions	8 (5.7)	10 (7.2)	1 (1.6)	7 (11.3)

## Discussion

### Principal Findings

Use of GenAI to respond to patient messages on behalf of PCPs is promoted as a promising tool for PCPs, but much remains unknown about the quality of the communication contained in the drafts and where the opportunities are for improvement. Applying a medical communication framework to assess GenAI responses, we observed that while communication standards were often met successfully, they were also often compromised. As a strength, GenAI responses contained many expressions of respect and facilitated appropriate next steps. However, there were also notable limitations in communication standards, including asking appropriate follow-up questions, matching information delivery to the patient’s informational need, and recognizing and responding to emotion. We draw several insights from these observations.

In our study setting, the GenAI model had limited access to chart information (eg, medications but not clinic notes) and responded only in the context of patient-PCP communication, and prompts were optimized through multiple rounds of robust prompt engineering. Our results suggest that, in that setting, GenAI responses may not only have many strengths but also many limitations when responding to real primary care patient messages. In many prior tests of GenAI responses, often with favorable assessments of GenAI performance, patient-generated content was processed and formatted for input into the model [25-27]. In primary care settings, patient messages to their providers are often more complex, containing multiple questions and nuances, and picking up from prior conversations that may or may not be documented. Clinician judgment is important for discerning and navigating the context, patients’ emotions, their informational needs, and appropriate follow-up questions. GenAI drafts in their current form may be inadequate for such tasks, in part, because of incomplete information and also because the output is a product of language prediction rather than true reasoning. Thus, GenAI drafts may often contain useful portions such as expressions of respect or explanation of common next steps, but our results suggest that it would be common to mishandle other aspects of communication important for patient care within the same draft.

Our observations align with others’ reports of GenAI draft evaluations in real health care settings. In one study, physicians rated GenAI responses more highly in communication quality than actual responses, likely reflecting GenAI strengths in rapport-building statements [17]. In another study, actual physician responses were rated higher in informational and emotional aspects than GenAI responses to negatively charged patient messages [28], likely reflecting limitations in judicious information delivery and responding to emotion. Other investigators observed that when GenAI responses to hypothetical patient questions had the potential for harm, it was because of inadequate assessment or communication of the acuity of the clinical scenario [29], reflecting that the limitation in judgment seemed to underlie the limitations we observed in our study.

Notably, our results also reiterate that GenAI draft usage does not imply high draft communication quality. Reviewers in our study indicated that they would use almost 60% of GenAI drafts, although 73% of drafts had at least 1 limitation in communication. On one hand, this may suggest that despite their limitations, GenAI drafts have some utility for busy clinicians. On the other hand, the use of drafts that fall short of communication standards could introduce various risks, such as anchoring or automation bias [29]. Careful proofreading is needed to identify potential misinformation [30]. Reading, fact-checking, and revising GenAI drafts may introduce new cognitive burdens and could explain why objective measures of clinician EHR usage have not been impacted by introducing GenAI draft support [14,15] and why physicians were not likely to recommend the tool to others [16]. Future studies of the impact of GenAI tools on cognitive load are needed.

Our exploratory analysis suggests that some limitations, at least in information delivery, could be overcome by providing more comprehensive access to information in the chart, including notes and other messages. Improvements might also be achieved by providing the model with more granular information about local practices and policies to better direct patients. However, those marginal benefits would have to be weighed against the costs of continuous prompt engineering, including supporting local prompt engineers and charges per token of model input and output. Other reasons we identified for limitations in information delivery, such as failure to address the essence of the patient’s question or the information being wrong, would be difficult to correct. Future advancements in large language model technology, and use of models that are trained specifically using health care communication data, may mitigate current limitations.

Given some of the observed limitations of GenAI responses, there are several potential ways forward for using the current technology in primary care. One is to restrict application to specific contexts or tasks, or for a narrower scope of questions. In an application of this tool in urology, drafts were more likely to be rated as acceptable by urologists when the question was “easy,” compared with questions that were deemed “difficult” (47% vs 34%) [31]. Better identifying primary care messages appropriate for GenAI response may improve performance; it is important to note that in our study, GenAI performance was similar between clerical and general patient messages, at least as categorized by the vendor, suggesting that alternative classification schemes would be needed to identify messages appropriate for GenAI response. Another option may be to apply GenAI as a proofreading tool, rather than a draft-generating tool, so that it improves the provider-drafted messages for readability, professionalism, or other aspects of communication. A third option may be to have patients self-select questions to ask GenAI versus PCPs, so that patients format questions de novo for input into GenAI, and responses do not suffer from lack of contextual information. Organizations will have to weigh the potential net benefit of these approaches with other potential solutions, such as investing in robust best practices for inbox management [8] or hiring dedicated clinical staff for inbox management.

### Strengths and Limitations

Our report has several key strengths. We used a well-accepted “best practice” medical communication framework to assess strengths and limitations, which to our knowledge is a novel application for assessing GenAI. While regulatory oversight of GenAI tools is urgently needed [32], clinical standards must also be used to evaluate and shape their impact [33], and our efforts align with that paradigm. We focused on messaging between patients and PCPs specifically, which is important because prior studies suggest that use and perceptions of GenAI responses differ by specialty and health care roles [14,16]. We assessed GenAI responses to real patient messages, rather than priming them for GenAI response, and those patient messages were purposively sampled to maximize variation in the sample and thus expose both strengths and limitations of the model. The GenAI drafts we assessed benefited from multiple prior rounds of prompt engineering, ensuring that the GenAI output we evaluated had been carefully tailored for the task of responding to primary care messages as PCPs. This tailoring may explain why the draft usage rate in our study was so high, compared with 12%‐20% reported in other studies [14,16].

There are also important limitations to our work. This represents the experience of a single institution applying GenAI in a specific context, for example, for messages between patients and PCPs with limited chart information, and experiences may differ with newer GPT models and other applications and contexts, including specialty care and inpatient versus emergency care settings. Patient messages to only 6 PCPs were included in the study, and the sample of 201 GenAI drafts could be considered small; however, we maximized variation within the sample as described, which other studies have not done, and our sample size is similar or larger than published studies on this topic (50‐175 GenAI messages) [17,28]. Another limitation is that we used a specific medical communication framework, and themes and constructs may have differed with the application of a different framework. We believe that our selection was justified because the “best practice” framework we used synthesizes many common communication frameworks. The same constructs in the “best practice framework, which addresses traditional face-to-face patient-physician encounters, are also applicable to inbox communication [34,35]. Some improvement in GenAI responses might have been achieved if the model were provided greater access to contextual clinical information beyond the limited elements used in our model, such as chart notes. However, as described, such access was limited by institutional data privacy policies, costs, and mode interval time limits, and these constraints are likely to be common to many other institutions. Finally, the tool we piloted was based on GPT 3.5; advances in the model are likely to overcome some of the limitations we observed.

### Conclusions

Our results suggest when GenAI was used to reply to patient messages on behalf of PCPs, with limited access to chart information and after robust rounds of prompt optimization, communication standards were often met and often compromised. GenAI response strengths were in making polite statements and outlining common next steps. However, frequently observed limitations were related to communication domains requiring clinician judgment, namely, asking appropriate follow-up questions, providing the right information for patients’ informational needs, and recognizing and responding to emotions. Overall communication quality seems unpredictable. These substantial limitations may limit GenAI draft utility.

## Supplementary material

10.2196/71966 Multimedia Appendix 1 Observed frequency of strengths and limitations across communication domains for all messages (n=201).

## Abbreviations

EHR: electronic health record
GenAI: generative artificial intelligence
PCP: primary care physician and advanced practice provider
