# Supplementary figures and images for: Use of a Medical Communication Framework to Assess the Quality of Generative Artificial Intelligence Replies to Primary Care Patient Portal Messages: Content Analysis
Source: JMIR Form Res. 2025 Jul 31;9:e71966. doi: 10.2196/71966 (PMC12313158; doi:10.2196/71966)

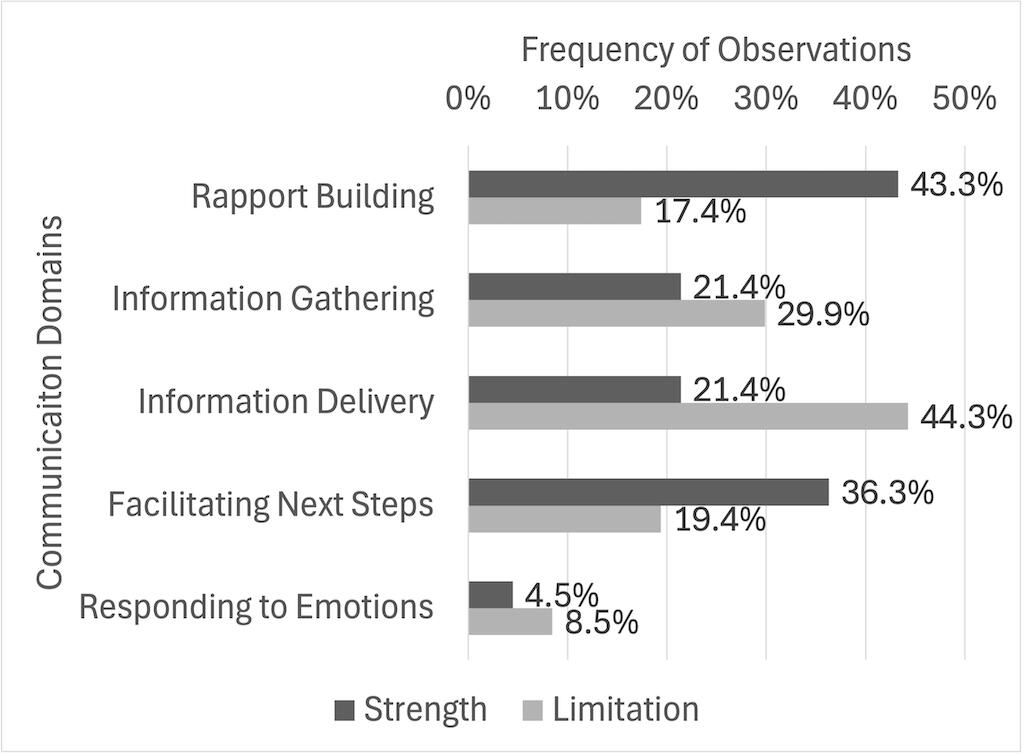

Supplement: Multimedia Appendix 1 [file formative-v9-e71966-s001.jpg]
